# Motor learning characterization in people with autism spectrum disorder: A systematic review

**DOI:** 10.1590/1980-57642016dn11-030010

**Published:** 2017

**Authors:** Íbis Ariana Peña de Moraes, Thais Massetti, Tânia Brusque Crocetta, Talita Dias da Silva, Lilian Del Ciello de Menezes, Carlos Bandeira de Mello Monteiro, Fernando Henrique Magalhães

**Affiliations:** 1 Post-graduate Program in Sciences of Physical Activity – School of Arts, Sciences and Humanities – University of São Paulo, São Paulo, SP, Brazil.; 2 Post-graduate Program in Rehabilitation Sciences – Faculty of Medicine – University of São Paulo, São Paulo, SP, Brazil.; 3 Department of Morphology and Physiology – Faculty of Medicine of ABC – Santo André, SP, Brazil.; 4 Post-graduate Program in Cardiology – Federal University of São Paulo – Paulista School of Medicine – São Paulo, SP, Brazil.

**Keywords:** motor learning, motor skill, motor control, performance, aprendizagem motora, habilidade motora, controle motor, desempenho

## Abstract

Autism Spectrum Disorder (ASD) is a neurodevelopmental disorder primarily characterized by deficits in social interaction, communication and implicit skill learning.

**OBJECTIVE:**

To analyse the results of research on "motor learning" and the means used for measuring "autistic disorder".

**METHODS:**

A systematic literature search was done using Medline/PubMed, Web of Science, BVS (virtual health library), and PsycINFO. We included articles that contained the keywords "autism" and "motor learning". The variables considered were the methodological aspects; results presented, and the methodological quality of the studies.

**RESULTS:**

A total of 42 studies were identified; 33 articles were excluded because they did not meet the inclusion criteria. Data were extracted from nine eligible studies and summarized.

**CONCLUSION:**

We concluded that although individuals with ASD showed performance difficulties in different memory and motor learning tasks, acquisition of skills still takes place in this population; however, this skill acquisition is related to heterogeneous events, occurring without the awareness of the individual.

## RESUMO

O Transtorno do Espectro Autista (TEA) é uma desordem do desenvolvimento neurológico caracterizada principalmente por déficits na interação social, comunicação e aprendizagem de habilidades implícitas.

**OBJETIVO:**

Analisar os resultados da pesquisa sobre "aprendizagem motora" e suas formas de mensurar na "desordem autista".

**MÉTODOS:**

Uma pesquisa bibliográfica sistemática foi feita usando Medline/PubMed, Web of Science, BVS (biblioteca virtual de saúde) e PsycINFO. Incluímos artigos que mostraram as palavras-chave "autismo" e "aprendizagem motora". As variáveis consideradas foram os aspectos metodológicos; os resultados apresentados e a qualidade metodológica dos estudos.

**RESULTADOS:**

Um total de 42 artigos foram identificados; foram excluídos 33 artigos porque não atendiam aos critérios de inclusão. Os dados foram extraídos de nove estudos elegíveis e resumidos.

**CONCLUSÃO:**

Concluímos que, embora as pessoas com TEA tenham demonstrado dificuldades de desempenho em diferentes tarefas de aprendizagem motora de memória, a aquisição de habilidades ocorre nessa população; No entanto, essa aquisição de habilidades está relacionada a eventos heterogêneos, ocorrendo sem a consciência dos indivíduos.

## INTRODUCTION

Autism spectrum disorder (ASD) is a heterogeneous neurodevelopmental disorder of multifactorial origin, characterized by deficits in social interaction and communication;^1^ however, motor coordination deficits are increasingly recognized as a prevalent feature of these conditions.^2^ Today, ASD is generally incurable, although it is treatable to a variable degree to prevent worse outcomes.^3^ Therefore, continued efforts should be made to promote the early identification of children with ASD so that interventions can be started at the youngest possible age.

The diagnosis of ASD has systematically risen since Kanner's description in 1943 and Asperger's definition in 1944. An increase in numbers has met with an increase in litigation regarding ASD and the Individuals with Disabilities Education Improvement Act.^4^ The prevalence of 4-year-old ASD was 13.4 per 1,000, which was 30% lower than the prevalence of ASD at 8 years of age, suggesting a progression in reducing the age of the first ASD assessment.^5^

The frequency of ASD diagnoses has been increasing for decades, but researchers cannot agree on whether the trend is a result of increased awareness, improved detection, expanding definitions, an actual increase in incidence, a combination of these factors,^6^ or the complex nature of these overlapping disorders and changes in clinical definitions over time.^7^

Predominantly, children with ASD have difficulty performing skilled movements^8^ and show a host of motor disorders including poor coordination, poor tool use, and delayed learning of complex motor skills.^9^

ASD, which has seen an alarming rise in prevalence, is a common condition that family physicians encounter in the clinical setting.^10^ The revision of diagnostic criteria for ASD has been widely anticipated and is expected to be an important contribution to the refinement of the definition of ASD. In the upcoming DSM-V (Fifth Edition of the Diagnostic and Statistical Manual of Mental Disorders), several changes have been made compared with the previous diagnostic criteria.^11^ The new definition has four different domains with sub-criteria for diagnosis. It uses one "umbrella" term of ASD with different levels of severity and no longer uses terms such as Asperger syndrome, classic autism, or pervasive development disorder. It has no emphasis on language delay and age of onset, only that ASD is defined as a neurodevelopmental disorder with symptoms in early childhood, although the disorder may be first diagnosed later in life.^10^

The three areas of impairments in ASD have been reduced to two areas; namely, a social-communication domain and a behavioral domain, including fixated interests and repetitive behaviors. In addition, the clinical presentation of ASD is described in more detail in terms of clinical specificity.^11^

Besides an adequate diagnosis, appropriate behavioral therapy and rehabilitation treatment significantly affect the prognoses and, thus, it is important to confirm an early diagnosis and to treat the disorder at an early stage. Treatment is more effective when persons can learn and properly perform the proposed task. Based on this evidence, some authors study the occurrence of motor learning in ASD from their practice.^12^

In addition, parents and clinicians frequently observe children with ASD displaying a clumsy gait, poor muscle tone, imbalance, as well as poor manual dexterity and coordination. Considering the consistent clinical reports of impaired motor functioning in ASD, motor examination may provide a window into the underlying neurobiological substrate of the disorder. Motor signs may serve as markers for deficits in parallel or neighbouring brain systems important for the control of socialization and communication.^13^ Measures of motor function tend to be more overtly observable than measures of more complex social and behavioral systems.^8^

Theory suggests that one of the crucial steps in motor learning is the ability to form internal models: i.e., to predict the sensory consequences of motor commands and learn from errors to improve performance on the next attempt.^9^ Effective development of praxis involves connections between multiple brain regions. The angular and supramarginal gyri are thought to be the site of storage of learned time-space movement representations or "action sequences". It is thought that these movement representations help to program the premotor cortex, which is involved in transcoding them into motor programs that, in turn, activate the motor cortex for execution.^8^

Thus, the cerebellum appears to be an important site for the acquisition of internal models and, indeed, the development of the cerebellum is abnormal in children with ASD.^9^ In particular, numerous histopathological and magnetic resonance imaging studies have reported reduced volume in the fastigial nuclei and cerebellar vermis lobules VI–VII in children with ASD. This is a region supporting ocular motor function, verbal working memory, and speech coordination.^2^

Another change commonly found in ASD is motor behavior, but the etiology remains unclear.^14^ Deficits in imitation have also been emphasized in the ASD literature, as well as abnormalities in "self-other mapping" (thought to be associated with dysfunction within mirror neuron systems) and have been hypothesized as contributing to impaired development of empathy, joint attention, and theory of mind.^8^

Studies of children with ASD reveal anomalous patterns of motor learning and impaired execution of skilled motor gestures. These findings robustly correlate with measures of social and communicative function, suggesting that anomalous action model formation may contribute to impaired development of social and communicative (in addition to motor) ability in ASD.^15^ Careful investigation of motor adaptation may therefore provide insight into the neurological mechanisms contributing to impaired motor function and skill acquisition in these children.^9^

According to Larson et al.,^9^ more complex tasks which involve learning new tools for controlling or adapting the movements of the arm in response to a change of visual information can be difficult for persons with ASD. These include some tasks in which disturbances are purely visual, rather than mechanical, and the individuals tested have never experienced any of these uncommon disturbances.^9^

The studies of Jones et al.^16^ showed that children with ASD consistently experience difficulty learning an abstract rule from a discrete physical reward. Rule learning is facilitated by the provision of more concrete reinforcement, suggesting an underlying difficulty in forming conceptual connections. It is important to identify why persons with ASD might have difficulty in learning and applying rules. One possibility is a disruption of the ability to learn from particular types of rewards. Most rule-learning paradigms involve the provision of feedback, either concrete and/or abstract.

The brain builds an association between action and sensory feedback to predict the sensory consequence of self-generated motor commands. This internal model of action is central to our ability to adapt movements and may also play a role in our ability to learn from observing others.^17^ With regard to the motor difficulties in ASD, some systematic reviews were conducted to show important developments in research for clinical practice and knowledge of ASD about motor learning, such as the improvement that child's play can have on executive function-related behaviors,^18^ the assessment of motor abilities in ASD using a computational context,^14^ or the identification of ability and disability in ASD.^19^

However, motor learning trials in this population are required due to a large discrepancy in the information. Systematic reviews are needed to provide up-to-date information on ASD and motor learning. Therefore, to support clinical need and bridge the knowledge gap, the aim of this study was to analyze the results of studies on motor learning in persons with ASD using a systematic review strategy.

## METHODS

This review was based on a systematic search of published articles available through April 2016 and conducted according to the Preferred Reporting Items for Systematic Reviews and Meta-Analyses guidelines.^20^ The study was recorded in the International prospective register of systematic reviews under number 46990.

### Eligibility criteria.

To increase confidence in the selection of articles, all potentially relevant articles were reviewed independently by two researchers, who reached consensus to establish which articles fulfilled the inclusion criteria.^21^

Abstracts of identified articles were then screened for the following inclusion criteria: [1] population included had a diagnosis of ASD; and [2] motor learning was assessed in this population. There were no restrictions on minimum sample size. Articles were excluded if they were: [1] not data-based (e.g., books, theoretical papers, or secondary reviews); [2] studies not published in English; [3] examined populations not explicitly identified as having a diagnosis of ASD; or [4] did not include motor learning information.

All identified studies were grouped in EndNote Web (Thomson Reuters) and duplicates removed.

### Information sources and search.

The current systematic review was based on published articles available through April 2016. The search was conducted on the databases PubMed (http://www.ncbi.nlm.nih.gov/pubmed), Web of Science (https://isiknowledge.com), BVS (virtual health library; http://bvsalud.org), and PsycINFO (http://www.apa.org/pubs/databases/psycinfo/) which were searched concurrently for entries using the established keywords. We included articles that contained both the keywords "motor learning" AND "autistic disorder" OR "autism".

The article search used the terms: "autistic disorder" OR "autism" AND "motor learning", "autistic disorder" OR "autism" AND "motor control", "autistic disorder" OR "autism" AND "performance", "autistic disorder" OR "autism" AND "motor skill"; surveyed studies that contained this combination of words anywhere in the text were selected.

**Figure 1:**
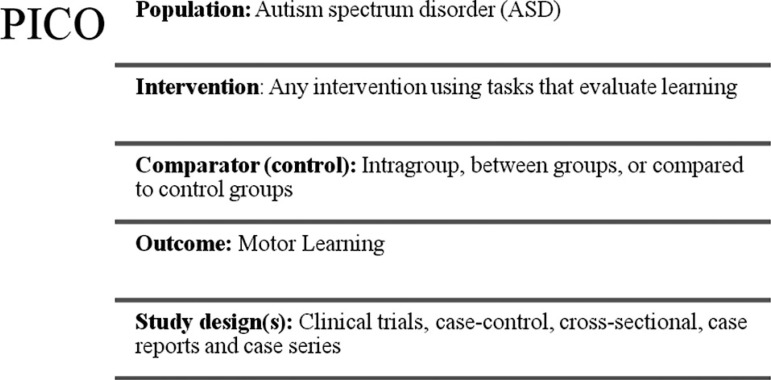
Representation of the search strategy – according to the Population Intervention Comparison Outcome (PICO) strategy.

Finally, reference lists of retrieved studies were hand-searched in order to identify any additional relevant studies. Key words and combination of key words were used to search the electronic databases and were organized according to the Population Intervention Comparison Outcome (PICO) strategy. As performed by Figoni^22^ and Massetti et al.,^23^ we used the search strategy based on their composition according to the PICO method, to locate and compare different studies (Figure 1). In this model, the search strategy was based on the topics: population (P), intervention (I), control group (C), and outcome (O) and on several searches in the aforementioned databases.

We used 3 steps to select the articles. The first step was to look for articles in databases and read their titles and abstracts. The second step was the exclusion of studies by title, abstract, or our inclusion criteria. The third and final step was to analyse the eligible studies.^23^

### Study selection and data collection processes.

After performing the initial literature searches, each study title and abstract was screened for eligibility by the first author. Full texts of all potentially relevant studies were subsequently retrieved and further examined for eligibility. The Preferred Reporting Items for Systematic Reviews and Meta-Analyses guidelines flow diagram (Figure 1) provides more detailed information regarding the selection process of studies.

To increase confidence in the selection of articles, all potentially relevant articles were reviewed independently by two researchers. After reading all potential studies, the researchers reached consensus to establish which articles fulfilled the inclusion criteria.^21^

### Analysis.

Motor learning is a phenomenon that refers to relatively permanent neuromotor changes, leading to the acquired ability to perform motor skills. Such changes ensure that the appropriate movement is achieved. They are derived from experience and practice, resulting in the acquisition, retention, and transfer of motor skills.^24^ However, motor learning can be measured by improvements in performance, which can be seen to increase and correct errors of execution and decrease duration of the task. In a developmental context, impaired performance of skilled gestures may be secondary to a fundamental problem with acquiring motor skills.^9^

Motor learning is the study of the acquisition of motor skills or the performance improvement of learned or highly practiced motor skills.^25^ However, this changing capability in motor learning is not directly measurable, because the changes responsible for motor learning are complex processes within the central nervous system. Therefore, change can be inferred by sustained improved performance^26^ and also individual differences in repetitive behaviours within the ASD may affect which areas of the brain are engaged during motor learning.^27^

Traditionally, therapists often use rational arguments, many verbal instructions, and available sensory feedback or feedback from an external source to engage patients in motor learning.^28^^,^ 29

Acceptable performance of a motor skill within a single session (or series of sessions) does not demonstrate that the skill has been learned. A skill is not considered truly learned until retention and/or transfer of that particular skill is demonstrated. These skills are classified by: [1] size of the movement – gross or fine motor skills; [2] beginning and end points – continuous rhythmicity; and [3] characterizing the stability of the environment in which the task is being performed – open or closed (temporal and spatial features of the environment where a task is performed.^30^

Practice which leads to optimal learning depends on the task being learned and the characteristics of the learner (e.g., age, stage of learning). The best practice design will not simply promote immediate performance effects, but, more importantly, will promote long-term learning.^31^

## RESULTS

Nine articles met the inclusion criteria for this review. The overall results for the studies included in this review are briefly presented below in Table 1.

**Table 1:** Detailed representation of studies.

Study reference	Participants	Subject characteristics	Outline of experimental Methodology relevant to this review	Principal outcomes	Oxford evidence
(Marko et al., 2015)	40 children	Aged between 8 and 12 years **20 – ASD** **20 – TD**	Changes in measurements of the volume of brain structures may be related to the learning patterns in relation to errors when analysed by a motor learning task in which reaching movements were affected.	The increased sensitivity to proprioceptive error and a decreased sensitivity to visual error may be associated with abnormalities in cerebellum volume.	**2C**
(Izadi-Najafabadi et al., 2015)	62 children	Aged between 7 and 11 years **30 – ASD** **32 – TD**	This was the first study to evaluate both implicit and explicit motor learning using the same task which makes it possible to compare these two types of learning. For this, a version of the SRTT that alternates between repeating and random events was used; the test was able to hide the sequence and track the sequence-specific learning.	Implicit motor learning is not affected in these children. Applying the findings of this study to cognitive orientation to daily occupational performance approach, the impaired explicit motor learning through the global strategy ''Plan'' and domain specific strategies can be supported by implicit approaches.	**3B**
(Travers et al., 2015)	30 children and adults	Aged between 17 and 23 years **15 – ASD** **15 – TD**	Recent behavioural studies on ASD and TD have shown similar motor learning in both groups. However, this study showed that individuals with ASD had less activation in areas associated with implicit learning because of possible explicit learning compensation.	Individuals with ASD who had more severe repetitive behaviour/restricted interest symptoms demonstrated greater decreased activation in the right SPL and right precuneus regions during motor learning which can thus play an important role in motor learning and repetitive behaviour in individuals with ASD.	**3B**
(Johnson et al., 2013)	36 children	Aged between 9 and 14 years **10 – HFA** **13 – ASD** **12 – TD**	This study contributes to a growing body of evidence centrally implicating the cerebellum in motor dysfunction in autism. HFA and ASD have overlapping etiologies, although they show distinctions that extend beyond their present diagnostic criteria of language and cognitive delay.	This study makes an important contribution to our understanding of the nature of motor function, highlighting deficits in processing visual feedback and motor learning. These deficits were greater in HFA than in ASD.	**3B**
(Izawa et al., 2012)	60 children	Aged between 9 and 12 years **23 – ASD** **17 – ADHD** **20 – TD**	It remains unclear whether the anomalous pattern of motor learning is specific to autism. For this purpose, the authors sought patterns of generalization in motor learning, comparing individuals with ASD, TD and individuals with attention deficit hyperactivity disorder (ADHD), because the latter also have a development disorder.	The findings suggest that there is a specific pattern of altered motor learning associated with autism. This is because the children with ASD show a slower rate of learning and an altered pattern of generalization that is predictive of impaired motor, imitation, and social skills.	**2C**
(Dowell et al., 2009)	87 children	Aged between 8 and 13 years **37 – ASD (HFA or AS)** **50 – TD**	Dyspraxia in autism seems to be associated with poor training of spatial representations as well as transcoding and execution. Abnormality distributed through parietal pre-motor, and a motor circuit, and the abnormal connectivity may be implicated.	Surprisingly, children with autism did not show impaired performance on the transitive gestures score of the postural knowledge test (although there appears to be a statistical trend toward children with autism demonstrating worse recognition of transitive gestures); a lack of statistical power may be a contributing factor to this finding.	**2C**
(Gidley Larson et al., 2008)	41 children	Aged between 8 and 13 years **21 – HFA** **20 – TD**	Sparse adaptation suggests that alternate mechanisms contribute to the development of motor skills in autism. In addition, the findings may have therapeutic implications, especially for a reliable mechanism by which children with autism can more effectively change their behaviour.	Children with autism demonstrated normal motor adaptation in a number of tasks that required acquisition of an internal model.	**4**
(Gidley Larson and Mostofsky, 2008)	153 children	Aged between 8 and 13 years **52 – HFA** **39 – ADHD** **62 – TD**	Mechanisms underlying acquisition of novel movement patterns may differ in children with autism. The findings may help explain impaired skill development in children with autism and guide approaches for helping children learn novel motor, social, and communicative skills.	Children with HFA showed differences in the pattern of visuomotor sequence learning and these differences persisted even after minimizing individual differences in motor execution. Evidence for specificity of this impairment is demonstrated in that children with ADHD did not show differences in the pattern of motor learning compared with a group of TD children. Detailed analysis revealed that while children with HFA showed similar gains across blocks of trials, they failed to show an expected decline in performance when an interfering pattern was introduced.	**2B**
(Wek and Husak, 1989)	8 children	Aged between 8 and 13 years **8 – ASD**	Further research on the applicability of motor learning principles for special populations should be carried out using larger groups.	Rotary pursuit tracking by autistic children does not appear to be identical to that of children without disabilities. As a result, traditional findings from the literature on massed and distributed practice are not totally applicable. Further research on the generalizability of motor learning principles to special populations is encouraged.	**4**

ASD: Autism spectrum disorder; TD: Typical development; HFA: High-functioning autism; ADHD: Attention deficit hyperactivity disorder; AS: Autism spectrum; SRTT: Serial Reaction Time Task.

### Selection of studies.

**Figure 2:**
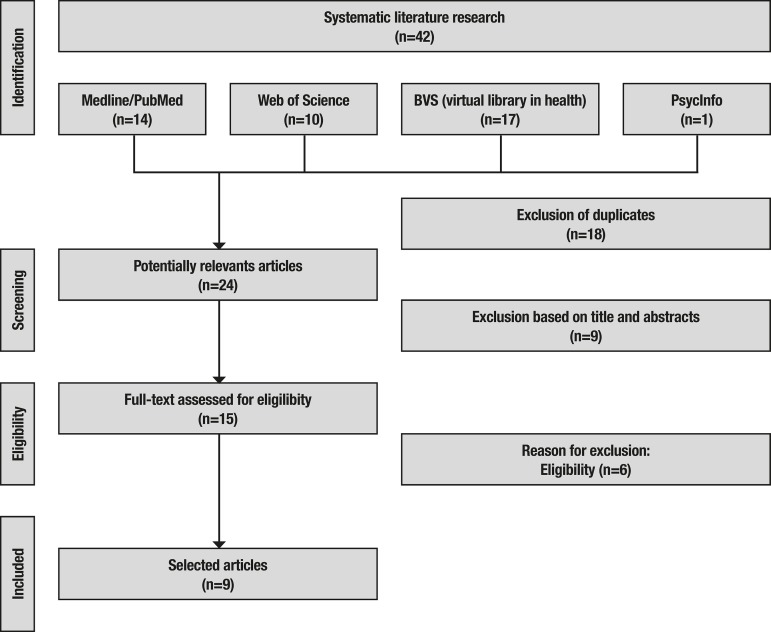
Flowchart of selected articles adapted from Moher et al. (2009).

The databases searched were Medline/Pubmed, Web of Science, BVS (virtual health library), and PsycINFO. Two reviewers screened all abstracts and full-text articles independently. The initial search carried out on four databases identified 42 articles that were evaluated according to the inclusion criteria. Nine studies met the eligibility criteria and were analysed in the systematic review. This review identified 42 studies (PubMed n=14; Web of Science n=10; BVS n=17; and PsycINFO n=1) after the initial search of the databases. Consequently, 15 studies were analysed according to eligibility criteria; six studies were excluded, mostly for eligibility reasons. In this process, nine experimental studies fully met the eligibility criteria (Figure 2).

### Study characteristics.

More information on the methodological and general characteristics of all the analysed studies can be found in Table 1.

### Countries in which the data was collected.

The reviewed studies were carried out in Baltimore,^8^^,^ 9 ^,^ 17 ^,^ 32 ^,^ 33 California,^34^ and Alabama^27^ in the United States of America; Monash^2^ in Australia; and Najafab^35^ in Iran.

### Participant characteristics.

The nine reviewed studies included a total of 517 participants; of these selected studies, only six provided gender information, with 31 women participating and 256 men. The articles by Gidley Larson et al.,^9^ Gidley Larson & Mostofsky,^32^ and Johnson et al.^2^ do not distinguish the gender of their participants. Participants in the sample had a diagnosis of ASD, High Functioning Autism, Asperger's syndrome, or were individuals with typical development, used as a control group.

The age of participants in the studies ranged from 7 to 23 years. In four of the included studies, the participants were individuals whose age ranged from 8 to 13 years old.^8^^,^ 9 ^,^ 32 ^,^ 34 In one study, the participants were individuals between 9 and 12 years of age;^17^ another study included individuals aged between 9 and 14 years;^2^ the study by Izadi-Najafabadi et al.^35^ featured individuals aged between 7 and 11 years; those in the study by Marko et al.^33^ were aged between 8 and 12 years; and, in turn, the study with a sample of older individuals was that by Travers et al.^27^ with participants aged between 17 and 23 years.

The main limitations of these studies were the use, in certain studies, of cognitive justification for the limitations encountered in the execution of motor learning, but not for the deficit in motor behavior.

Another limiting factor was the very different measures and means of evaluating performance. The nature of the disease-matched individuals also affects the results of motor learning analysis. The studies included in this review have specific difficulties: small and heterogeneous samples. Few articles were included and not all were randomized controlled trials, recommended for evaluating the highest degree of evidence in systematic reviews.

## DISCUSSION

Motor deficiency in children with ASD has received increasing attention with abundant evidence clearly indicating the high prevalence of motor impairment. One of the major challenges in the management of motor skills in this population concerns the understanding of the task. However, before motor performance can be considered as a major feature of ASD, it is essential to establish a valid and reliable motor assessment for use in ASD children that incorporates appropriate adaptations to facilitate understanding of tasks and to ensure accurate assessment of motor performance.

ASD is a highly uncertain entity and little is stated about it with any degree of certainty. Understanding uncertainty and indeterminacy is important for an understanding of ASD and its heterogeneity.^36^ Over the past few years, awareness of motor problems in individuals with ASD has grown. Thus, a common feature is a deficiency of motor control and motor learning, a consequence for the majority of children with the disability.^37^

### Outcomes.

The main objective of this study was to analyse research findings about motor learning in persons with ASD. Having searched through different tests and tasks performed by subjects, the results show that there was a performance improvement during the stages of learning protocols.

Children with disabilities often have many disadvantages that have long-term consequences and motor problems place an additional burden on child and can significantly affect daily life and social interactions. Thus, determining the role that fine and gross motor skills can play in improving cognitive and social skills can help identify abilities that can be intervened early, which can reduce the impact of motor difficulties and may also help enable full potential.^38^

During tests aimed at analyzing motor learning from errors in visual feedback,^2^^,^ 9 ^,^ 33 it was noted that there is a major influence of cerebellar integrity in driving motor activities effectively. The cerebellum has historically been ascribed a role in motor learning and coordination and studies suggest that cerebellar changes may contribute to the range of cognitive, linguistic, social, and motor behaviours seen in ASD.^39^ In the study by Johnson et al.,^2^ evidence is shown for the contribution of the cerebellum in motor dysfunction in ASD, highlighting deficits in processing visual feedback and the learning engine. According to the author, these deficits were greater in High Functioning Autism than in ASD. Similar results were found in the study by Gidley Larson and Mostofsky^32^ comparing motor learning in ASD with attention deficit hyperactivity disorder patients through a constant and alternating speed task.

Other brain regions that showed decreased activity during the task were the right upper lobe and the right parietal precuneus. Activation in these areas is significantly related to behavioural learning during serial reaction time tasks. The results of the research Travers et al.^27^ indicate the presence, albeit less robust, of motor-linked implicit learning in ASD compared to individuals with typical development, indicating possible difficulties with motor learning in ASD.

The findings suggest that there is a specific pattern of altered motor learning associated with ASD; probably because people with ASD show a slower rate of learning and an altered pattern of generalization that is predictive of impaired motor, imitation, and social impairment.^17^ However, individuals with ASD demonstrated normal motor adaptation in a number of tasks that required acquisition of an internal model.^9^

### Learning motor characteristics and intervention protocol.

In this review, the number of participants with ASD in the nine studies ranged from 8 to 153. Even with a small sample size, considering the incidence of this disease, in eight of these studies there were control groups of children who were typically developing, and two studies compared ASD groups with groups of individuals with Attention deficit hyperactivity disorder. In the studies presented, the authors evaluated motor learning through questionnaires, tests, and different types of tasks, which analysed performance before and after, considering how the person has adapted the task through the analysis of the blocks during phases of the protocols followed. Johnson et al.^2^ used a saccade task adapted to verify motor learning in High Functioning Autism and ASD through the errors in visual feedback by means of a protocol with three phases: pre-adaptation, adaptation phase, and after fitting. The authors found that these deficits were greater in High Functioning Autism than in ASD, which provides a functional extension of current neuropathological evidence of greater disruption to the vermis lobules VI–VII and caudal fastigial nucleus, and/or the connections to and from these structures, in High Functioning Autism.

This study makes an important contribution to our understanding of the nature of motor function in these populations, highlighting deficits in processing visual feedback and motor learning. Therefore, it contributes to a growing body of evidence centrally implicating the cerebellum in ocular motor dysfunction in ASD. High Functioning Autism and ASD have overlapping etiologies, although both show distinctions that extend beyond their present diagnostic criteria of language and cognitive delay.^2^

The research of Dowell et al.^8^ used a questionnaire and motor tests and showed that children with ASD demonstrate marked impairments in praxis examination, with deficits observed not only in the performance of gestures of imitation, but also gestures of command and gestures of actual tool use, showing that dyspraxia in ASD seems to be associated with poor training of spatial representations as well as transcoding and executing. Therefore, as the results of the study reported that dyspraxia in ASD cannot be entirely accounted for by impairments in basic motor skills, this suggests the presence of additional contributory factors.^40^^,^ 41

Gidley Larson et al.^9^ considered three well-studied motor adaptation protocols to test the ability of children with ASD to acquire internal models of action. Two experiments involved learning to control a novel tool (reach adaptation with a robotic arm), while the third involved learning to compensate for a transformation in the visual input. As evidenced by the after-effects of adaptation, the children with ASD improved their performances through the formation of internal predictive models, with rates of acquisition and forgetting that did not differ from typically developing children. This suggests that alternate mechanisms are contributing to the development of motor skills in ASD. In addition, the findings may have therapeutic implications, especially as a reliable mechanism by which children with ASD can more effectively change their behaviour.

A similar study performed by Marko et al.^33^ measured learning from error as a function of the sensory modality of that error and found that children with ASD outperformed typically developing children when learning from errors that were sensed through proprioception, but underperformed typically developing children when learning from errors that were sensed through vision. Contradicting previous studies, the authors suggested that the abnormal patterns of motor learning in children with ASD – showing increased sensitivity to proprioceptive error and decreased sensitivity to visual error – may be associated with abnormalities in the cerebellum.

Another predictor of difficulties in motor learning in ASD children was discussed in a study^12^ where perceptual and motor tasks measuring key aspects of motor behaviour were used to investigate whether visual processing speed or visually triggered or visually guided movement differs between ASD individuals with or without speech onset delay. They concluded that ASD subjects with and without speech onset delay differ in visually guided and visually triggered behaviour and showed that early language skills are associated with slower movement in simple and complex motor tasks. It can be seen that these movement skill differences become more obvious with increasing age, reflecting the limited opportunities of children with ASD to practice and improve their movement skills.^42^

In the same context, the study by Gidley Larson and Mostofsky^32^ examined motor learning in children with ASD compared with both a Typical Development control group and a clinical control group (Attention deficit hyperactivity disorder group). They found that only children with ASD showed similar degrees of learning (i.e., similar rates of improvement in-time-on-target) across the circular blocks when compared with Typical Development children. In addition, children with Attention deficit hyperactivity disorder did not show impaired learning on either task compared to TD children. This lack of difference suggests that children with ASD were able to learn the motor sequence. It suggests that mechanisms underlying acquisition of novel movement patterns may differ in children with ASD. These findings may help explain impaired skill development in children with autism and guide approaches for helping children learn novel motor, social, and communicative skills.

Wek and Husak^34^ performed a task during 10 consecutive days and showed that for persons with normal development, massed practice conditions resulted in poorer performance when compared with distributed practice, largely because of fatigue and the inability to process cognitive information in the short rest intervals (e.g. massed practice characteristic). However, the results showed that, for the ASD sample, there were no significant differences between the groups or across trials, since there was no effect on trials for the distributed practice group, where the scores of this group were similar to those of the massed practice group. It can be hypothesized that ASD had different adaptation mechanisms to fatigue and to process cognitive information. The authors suggested that further research on the applicability of motor learning principles for special populations should be carried out using larger groups.

The study by Minshew and Williams^43^ showed the change in thinking from regarding ASD as a disorder of regional brain dysfunction to a model of ASD as a large-scale neural systems disorder with alterations in cortical systems connectivity. This is due to developmental alterations in white matter and their role in intrahemispheric connectivity. Corroborating this, Mostofsky et al.^44^ demonstrated an association between increasing radiate white matter volume and functional impairment in children with ASD, which presented as basic motor skill impairment.

Another practical study was conducted by Allen and Courchesne^45^ that, through an analysis of functional magnetic resonance imaging during a motor task, concluded that individuals with ASD showed significantly greater cerebellar motor activation and significantly less cerebellar attention activation, suggesting that developmental cerebellar abnormality has different functional implications for cognitive and motor systems. In the study of Lang and Bastian,^46^ who analysed the role of the cerebellum in adapting anticipatory muscle activity during a multi-jointed catching task, the authors concluded that the cerebellum is important in generating the appropriate anticipatory muscle activity across multiple muscles and modifying it in response to changing demands though trial-and-error practice.

Corroborating these results, the study of Mostofsky et al.^44^ stated that cerebellar components have been implicated in contributing to the pathophysiology of ASD, but the results indicated that the group with autism had significant impairments in procedural learning compared with the control group. The data suggested that deficits in procedural learning may contribute to the cognitive and behavioural phenotype of ASD; these deficits may be secondary to abnormalities in cerebellar-frontal circuits. According to Muller et al.,^47^ another region apparently altered in ASD individuals is the primary and secondary motor cortex. The researchers stated that, for relatively complex functions of visuomotor learning, ASD subjects apply more stimulus-driven strategies to the digit-sequence task, even at later learning stages.

### Study limitations.

This study provides a systematic review and important guidelines for future research on intervention with regard to ASD and motor learning. However, future studies should clarify certain aspects about the study design and methodology, outcome measures, follow-up investigations, and transfer effects of the practice. Another limitation of our study was the fact that heterogeneity and comorbidities that might influence motor learning in ASD were not included in the review, representing topics of interest for futures studies.

We conclude that although individuals with ASD showed deficits in different tasks of memory and motor learning, acquisition skills still take place in this population; however, this skill acquisition is related to heterogeneous events, occurring without the awareness of the individual. Furthermore, the findings may have therapeutic implications through educational programs and more effective rehabilitation. Thus, further research is necessary; with an emphasis on experimentally controlled, blinded and randomized study designs, to better address motor learning in the population.
